# Biomineralization processes of calcite induced by bacteria isolated from marine sediments

**DOI:** 10.1590/S1517-838246220140533

**Published:** 2015-06-01

**Authors:** Shiping Wei, Hongpeng Cui, Zhenglong Jiang, Hao Liu, Hao He, Nianqiao Fang

**Affiliations:** China University of Geosciences, School of Marine Sciences, China University of Geosciences, Beijing, China, School of Marine Sciences, China University of Geosciences, Beijing, China.

**Keywords:** calcium carbonate precipitation, calcite, marine bacteria, urease

## Abstract

Biomineralization is a known natural phenomenon associated with a wide range of bacterial species. Bacterial-induced calcium carbonate precipitation by marine isolates was investigated in this study. Three genera of ureolytic bacteria, *Sporosarcina sp.*, *Bacillus sp.* and *Brevundimonas sp*. were observed to precipitate calcium carbonate minerals. Of these species, *Sporosarcina sp*. dominated the cultured isolates. *B. lentus* CP28 generated higher urease activity and facilitated more efficient precipitation of calcium carbonate at 3.24 ± 0.25 × 10^−4^ mg/cell. X-ray diffraction indicated that the dominant calcium carbonate phase was calcite. Scanning electron microscopy showed that morphologies of the minerals were dominated by cubic, rhombic and polygonal plate-like crystals. The dynamic process of microbial calcium carbonate precipitation revealed that *B. lentus* CP28 precipitated calcite crystals through the enzymatic hydrolysis of urea, and that when ammonium ion concentrations reached 746 mM and the pH reached 9.6, that favored calcite precipitation at a higher level of 96 mg/L. The results of this research provide evidence that a variety of marine bacteria can induce calcium carbonate precipitation, and may influence the marine carbonate cycle in natural environments.

## Introduction

Bacterial calcium carbonate precipitation is a biomineralization process, which is a common phenomenon in the bacterial kingdom (Boquet *et al.*, 1973). It can be achieved by two different mechanisms, as either biologically-controlled or biologically-induced mineralization (Mann, 1995). In biologically-controlled mineralization, the organisms, such as magnetotatic bacteria, diatoms and coccolithophores, use specific metabolic and genetic pathways to control the process (Bazylinski and Moskowitz, 1997). However, calcium carbonate precipitation by bacteria is generally regarded as induced mineralization, as the types of minerals produced are dependent on the environmental conditions (Brennan *et al.*, 2004). This phenomenon occurs worldwide with numerous bacterial species, in various environments, such as soils, freshwaters, oceans and saline lakes, found to participate in the precipitation of mineral carbonates (Douglas *et al.*, 1998; Rivadeneyra *et al.*, 1998; Peckman *et al.*, 1999; Zamarreño *et al.*, 2009). These bacteria play a fundamental role in the calcium biogeochemical cycle, which contributes to the formation of calcium carbonate sediments, deposits and rocks (Chafetz *et al.*, 1991; Paerl *et al.*, 2001).

Biologically-induced mineralization is usually carried out in open environments and the process is often linked to microbial cell surface structures and metabolic activities. Microbial extracellular polymeric substances (EPS) can trap and bind remarkable amounts of calcium to facilitate calcium carbonate precipitation, and most likely also play an essential role in calcium carbonate precipitation morphology and mineralogy (Arp *et al.*, 1999; Braissant *et al.*, 2007; Dupraz *et al.*, 2005). The mineralization process associated with microbial metabolic activities usually leads to an increase in environmental alkalinity, thereby facilitating calcium carbonate precipitation (Douglas and Beveridge, 1998; Castanier *et al.*, 1999). Among these metabolic activities, the most common is urea hydrolysis catalyzed by urease enzymes, which commonly occurs in large varieties of microorganisms (Mobley and Hausinger, 1989). The microbial urease enzyme hydrolyzes urea to produce carbonate and ammonia, increasing the pH and carbonate concentration, which then combines with environmental calcium to precipitate as calcium carbonate (Hammes *et al.*, 2003; Muynck *et al.*, 2010).

Calcite, aragonite and vaterite are three crystal polymorphs of calcium carbonate in bacterial systems, with calcite being the most common and stable bacterial carbonate polymorphs (Rodriguez-Navarro *et al.*, 2012). Bacterial mineralization of aragonite, often representing the metastable polymorph, has also been reported (Pedone *et al.*, 2010). The production of the polymorphs of calcite, aragonite and vaterite depend both on their growing environments and bacterial strains. It was reported that different bacteria precipitated different types of calcium carbonate and were mainly either spherical or polyhedral crystalline forms (Cañaveras *et al.*, 2001). Bacterial-induced carbonate minerals have often been reported in a large number of bacteria, such as cyanobacteria (Jansson and Northen, 2010), sulphate-reducing bacteria (Warthmann *et al.*, 2000), *Bacillus* (Goddette *et al.*, 1992; Betzel *et al.*, 1998; Jørgensen *et al.*, 2000), *Myxococcus* (Rodriguez-Navarro *et al.*, 2003; Gonzalez-Muñoz *et al.*, 2010), *Halobacteria* (S¢nchez-Rom¢n *et al.*, 2011) and *Pseudomona*s (Jha *et al.*, 2009). Groth *et al.* (2001) tested the crystal-producing ability among cave bacteria and found that all produced calcite except for *Bacillus sp*., which precipitated vaterites. Rodriguez-Navarro *et al.* (2003) reported that *M. xanthus* was able to induce precipitation of calcite and vaterite. Emerging evidence suggests that bacteria do not directly influence calcium carbonate morphology or polymorph selection (Chekroun *et al.*, 2004; Bosak *et al.*, 2005; Rodriguez-Navarro *et al.*, 2012). The morphological features instead may be influenced by the composition of the culture medium, the specific bacterial outer structures and their chemical nature, which might be crucial for the bacterial crystallization process (Gonzalez-Muñoz *et al.*, 2010). The aim of this study was to identify calcium carbonate producing bacteria in marine sediment and to characterize the CaCO_3_ crystals produced.

## Materials and Methods

### Bacteria isolation and culture conditions

Calcium carbonate precipitating strains were isolated from Beidaihe marine sediment (119°31′18.89″ N and 39°50′11.90″ E). The sample was suspended in a filter sterilized saline solution (0.85% NaCl), diluted appropriately and plated on calcium carbonate precipitation media (CCP) containing (per liter) 20 g of urea, 2.12 g NaHCO_3_, 10 g NH_4_Cl, 3 g of Nutrient broth, 30 mM CaCl_2_, 20 g agar, pH 8.5. The plates were then incubated at 28 °C for 7 days, and the appearing colonies were assessed under a stereomicroscope. The positive individual colonies were finally selected based on their visual crystal formation and purified by repeated streaking on the calcium carbonate precipitation media with CaCl_2_ removed.

### DNA extract, PCR amplification and sequencing

Bacterial genomic DNA was extracted from pure culture with the fast spin kit (Invitrogen) following the manufacturer's instructions. Amplification of 16S rRNA gene was performed in 50 μL of reaction mixture containing 0.25 mM each primer of 27f (5′-GTTTGATCCTG GCTCAG-3′) and 1492r (5′-TACCTTGTTACGACTT-3′), 0.2 mM dNTP, 1.5 mM MgCl_2_, 5 μL of Taq buffer, and 5 U Taq DNA polymerase (Invitrogen, USA), 10–20 ng template DNA. PCR was then performed on a thermalcycler under the following conditions: 95 °C for 5 min, 35 cycles of 50 s at 95 °C, 50 s at 45 °C and 1.5 min at 72 °C, followed by a final extension for 10 min at 72 °C. The PCR products were visualized on an agarose gel, and the bands with the corrected size were excised and purified using the Wizard SV gel purification protocol (Promega, USA). The partial 16S rRNA fragment was sequenced on an ABI 3730 automated DNA sequencer (Applied Biosystems).

### Phylogenetic analysis

Phylogenetic affiliation of each 16S rRNA sequence was initially queried by BLAST search to suggest the closest relatives against the GenBank database. The sequences were then aligned with their relatives using Clustal W, and phylogenetic trees were constructed from a matrix of pairwise genetic distances by the maximum-parsimony algorithm of the MEG 4 software. Three partial sequences of 16S rRNA genes from the strains, CP16, CP23 and CP28, isolated from Beidaihe marine sediment, have been deposited in the GenBank database under accession numbers: KF378645, KF378646, KF378647, respectively.

### Urease activity assay

All the isolates were tested for their urease activity on the urea agar media containing 1.0 g of pancreatic digest gelatin, 1.0 g of dextrose, 5.0 g of sodium chloride, 2.0 g of monosodium phosphate, 20.0 g of urea, 12.0 mg of phenol red, 15.0 g of agar, and the final pH was adjusted to 6.8 (Hammes *et al.*, 2003; Chahal *et al.*, 2011). 0.5 μL cell suspension of each candidate strain (10^6^ cells/mL) was inoculated on the urea agar media, and the plates were incubated at 28 °C for 1–2 days. The urease activity was resolved on the media to the extent of the indication of the pink-red color, which specifically represents the generation of alkaline conditions that are attributed to the production of ammonia via urease activity on urea. An *Escherichia coli* strain was chosen as the negative control.

### Test for calcium carbonate solubilization

Strains isolated from the calcium carbonate precipitation agar plates were tested for their solubilization capability of calcium carbonate on the media (CCS) containing (per liter) 0.5 g of yeast extract, 10 g of dextrose, 5 g of CaCl_2_, 0.5 g of (NH_4_)_2_SO_4_, 5 g of Ca_3_(PO_4_)_2_, 0.2 g of KCl, 0.1 g of MgSO_4_, 0.0001 g of MnSO_4_ and 0.0001 g of FeSO_4_, 20 g agar, pH 7.0, and grown at 28 °C for 5 days. The solubilization capability of calcium carbonate was quantified by measuring the diameter of the clear halo around a colony.

### Calcium carbonate precipitation and collection

For calcium carbonate precipitation and collection, bacteria were grown aerobically in 100 mL of liquid calcium carbonate precipitation media in 500 mL Erlenmeyer flasks and incubated at 28 °C for 60 h. The control consisted of uninoculated liquid calcium carbonate precipitation medium. At each time point and after the incubation, the whole culture was centrifuged at 10,000 g for 1 min. The pellet, which included calcium carbonate precipitate and the bacteria cells, was resuspended in 50 mL TE buffer (10 mM Tris, 1 mM EDTA pH 8.5). Lysozyme was added at a final concentration of 1 mg/mL and the cell suspension was incubated at 37 °C for 1 h to digest the bacteria cell wall. The cell debris was removed by centrifugation and the pellet was washed with sterile distilled water (pH 8.5), then air dried at 37 °C for 24 h. The pellet was weighed to estimate the amounts of carbonate crystals precipitated by the different strains and subjected to the following analyses.

### X-ray diffraction analysis (XRD)

X-ray diffraction (XRD) was used to determine the mineralogy of calcium carbonate precipitation induced by different bacteria. The collected dry precipitation of calcium carbonate was crushed using a mortar and pestle, then homogenized with ethanol. The powdered sample was back-packed into an aluminum sample holder and analyzed using XRD on a Panalytical X'Pert PRO MPD (Cu-K|Á) at the Nuclear Industry Geological Analysis and Testing Research Center (Beijing, China). Instrument parameters were set to 40-kV accelerating voltage and 35-mA current. Scans were run from 20° to 60° 2θ at a scanning speed of 0.01 °/s. The peak in the d (112) was used to determine the calcite minerals.

### Scanning electron microscopy (SEM)

Morphology of calcium carbonate precipitation was observed by scanning electron microscopy (SEM Hitachi S-450). The collected carbonate crystals were mounted directly into the SEM stubs and sputter-coated with a gold/palladium mixture (Hitachi HUS-5GB coating unit). Scanning was performed under the condition of accelerating voltage at 25 kV.

### Cell number, pH and chemical analytic methods

In order to determine the correlation of calcium carbonate formation to the parametric changes during the growth phase of *B. lentus* CP28, parameters such as cell number, pH and ammonia were monitored at constant time intervals. At each time point of post incubation, a 0.5 mL aliquot of the culture was taken from the flask, appropriately diluted, then spread on the nutrition broth agar (per liter, 5 g of enzymatic digest gelatin, 3 g of beef extract, 15 g of agar) and incubated at 28 °C for 24 h to determine the cell numbers. Calcium carbonate precipitation was determined as described above and the supernatant was used to determine the pH and the concentration of ammonia. pH was measured using a pH indicator (PB-10, Sartorius AG). Ammonia released in the medium as a result of urea hydrolysis was determined by the spectrophotometric method (Natarajan *et al.*, 1995).

## Results

### Isolation of bacteria involved in inducing calcium carbonate precipitation

Twenty strains were isolated from calcium carbonate precipitation agar plates, all of which could induce the precipitation of calcium carbonate under those conditions. Microscopy revealed that precipitation started with a scattered white spot circling the bacteria colony, then developed into a hard gray-white crystal covering the colony with an encircling scattered white spot appearing after 7 days. Based on the morphological differences of crystal formed on the agar plate, the strains were divided into two types, those demonstrating either strong induction or weak induction of calcium carbonate. Five and fifteen colonies, belonging to the two types respectively, were isolated and selected for the further studies.

### Phylogenetic analysis of the isolated candidate strains

**Figure 1:**
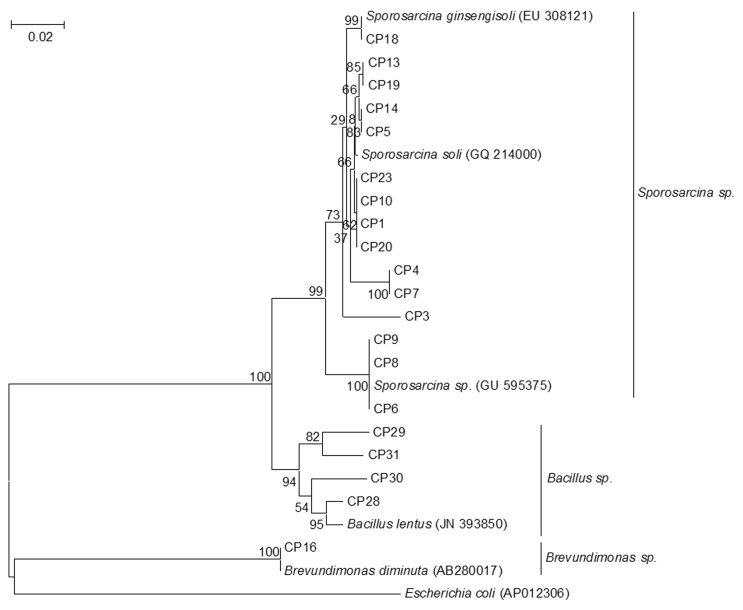
Neighbor-joining tree based on partial 16S rRNA gene sequences showing the phylogenetic relationship of the 20 isolates and their closest relatives. The phylogenetic tree was generated using approximately 1,400 bp of 16S rRNA sequence by the neighbor-joining method. Reference strains used in the tree can be retrieved with their accession numbers in GenBank. Scale bar equals approximately 2% nucleotide divergence.

A total of twenty isolates were identified and characterized by sequencing of 16S rDNA. These sequences were BLAST searched against the GenBank database using the BLASTN program. Twenty isolates belonging to three genera were identified, and had the closest relatives belonging to *Sporosarcina sp.*, *Bacillus sp.* and *Brevundimonas sp.* (Figure 1). Sequences related to *Sporosarcina sp.* (occupied 75% of total sequences based on 98% of sequence similarity) dominated the cultured isolates, which included 15 isolates (CP1, CP3 to CP10, CP13, CP14, CP18 to CP20, CP23), followed by *Bacillus sp.* (occupied 20% of total sequences based on 98% of sequence similarity), which included 4 isolates (CP28 to CP31) and *Brevundimonas sp.* (occupied 5% of total sequences based on 99% of sequence similarity) which was comprised of a single isolate (CP16).

### Characterization of three phylogenetic distinct strains

**Figure 2:**
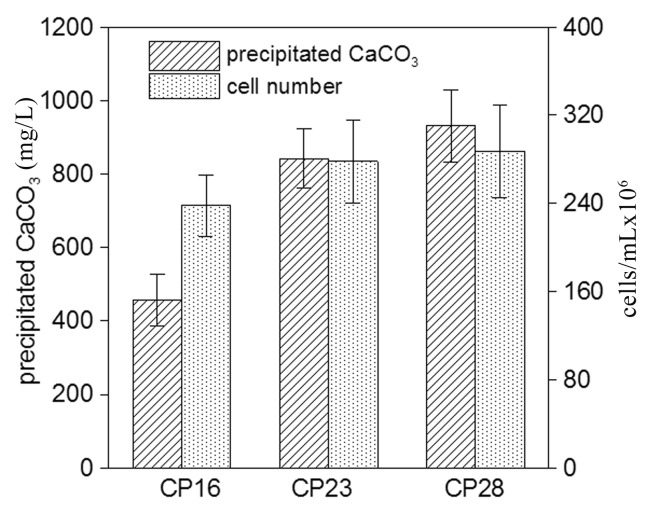
Comparison of the growth rate and capability of inducing calcium carbonate precipitation among the strains of *B. diminuta* CP16, *S. soli* CP23 and *B. lentus* CP28.

CP16, CP23 and CP28, closely related to *B. diminuta*, *S. soli* and *B. lentus*, were investigated and characterized by their growth rate, capability of inducing calcium carbonate precipitation, urease activity, and calcium carbonate solubilization ability. *B. lentus* CP28 grew faster and faciliated more calcium carbonate precipitation than the strains of *B. diminuta* CP16 and *S. soli* CP23 (Figure 2). After 60 h of incubation, the cell number of CP16, CP23 and CP 28 were 2.78 ± 0.38 × 10^6^, 2.38 ± 0.28 × 10^6^ and 2.87 ± 0.42 × 10^6^ cells/mL, respectively. The masses of the precipitates of the three strains were 842 ± 80, 456 ± 70 and 931 ± 98 mg/L, respectively. CP28 was the most efficient strain at inducing calcium carbonate precipitation when calculations were based on the mass of precipitation per cell, with CP28 capable of inducing calcium carbonate precipitation at 3.24 ± 0.25 × 10^−4^ mg/cell.

Microbial-induced calcium carbonate precipitation by urea hydrolysis was investigated extensively. The bacterium converts urea into ammonia by producing the enzyme urease, thus increasing the environmental pH and subsequently inducing calcium carbonate precipitation. All 20 of the isolated strains possessed the urease activity when tested in the urea agar media. Among of tested strains, CP23 and CP28 generated higher urease activity than CP16, whereas the *E. coli* strain did not show any purple color surrounding the inoculated site, which indicates a lack of urease activity. This urease activity assay result, together with the result in Figure 2, implied that the mass of calcium carbonate precipitation was directly linked to the urease activity, with higher urease activity causing more calcium carbonate precipitation. Therefore, strains CP16, CP23 and CP28 were chosen for further analyses.

CP16, CP23 and CP28 were dot inoculated on the calcium carbonate precipitation media and incubated for 7 days in order to compare the diameter of the crystal halo surrounding the inoculation site. Table 1 shows that diameter of the crystal ring formed by CP28 was larger than that of CP23. However, no calcium carbonate formed when plates were inoculated with *E. coli* control. To determine whether the isolates play roles in the deterioration of limestone by solubilizing calcium carbonate, carbonate-solubilization capability was tested on the calcium carbonate solubilization media. Both CP16 and CP23 dissolved calcium carbonate and formed a clear, circular halo around the inoculation site. However, CP28 did not form similar halos. These data suggested that the mechanism of calcium carbonate precipitation induced by bacteria may change with variations in environmental conditions.

**Table 1:** Characterization of the isolates on precipitation and dissolution of CaCO_3_.

Strains	CaCO_3_ precipitation on CCP media	CaCO_3_ dissolution on CCS media
*E. coli* CK	−^a^	−^a^
*B. diminuta* CP16	+	−^a^
*S. soli* CP23	+++	+
*B. lentus* CP28	++++	−^a^

Notes: + and −^a^ indicate the relative degree of precipitation or dissolution of CaCO_3_ among *E. coli* and the tested ureolytic bacteria.

### SEM and XRD analyses of microbically-induced calcium carbonate precipitation

**Figure 3:**
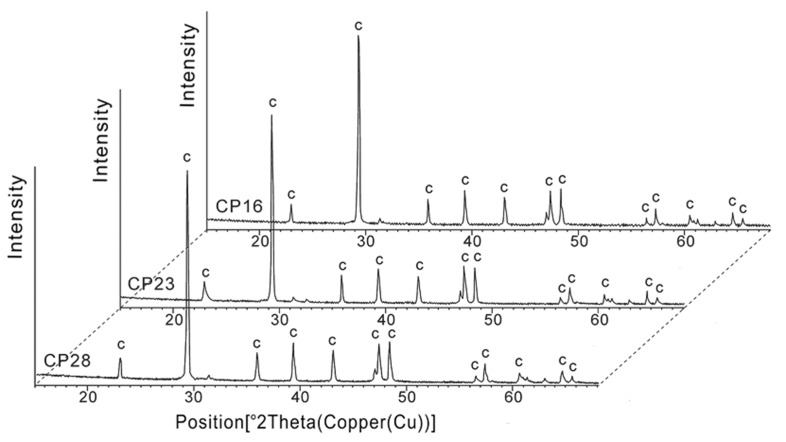
XRD spectra of the calcium carbonate crystals induced by bacteria. C, calcite. From top to bottom: *B. diminuta* CP16, *S. soli* CP23 and *B. lentus* CP28.

Precipitations collected on the 7th day were analyzed by XRD. The results showed that all three strains, *B. diminuta* CP16, *S. soli* CP23, *B. lentus* CP28, induced the formation of calcite (Figure 3), which was the only crystal assayed in the XRD spectra.

**Figure 4:**
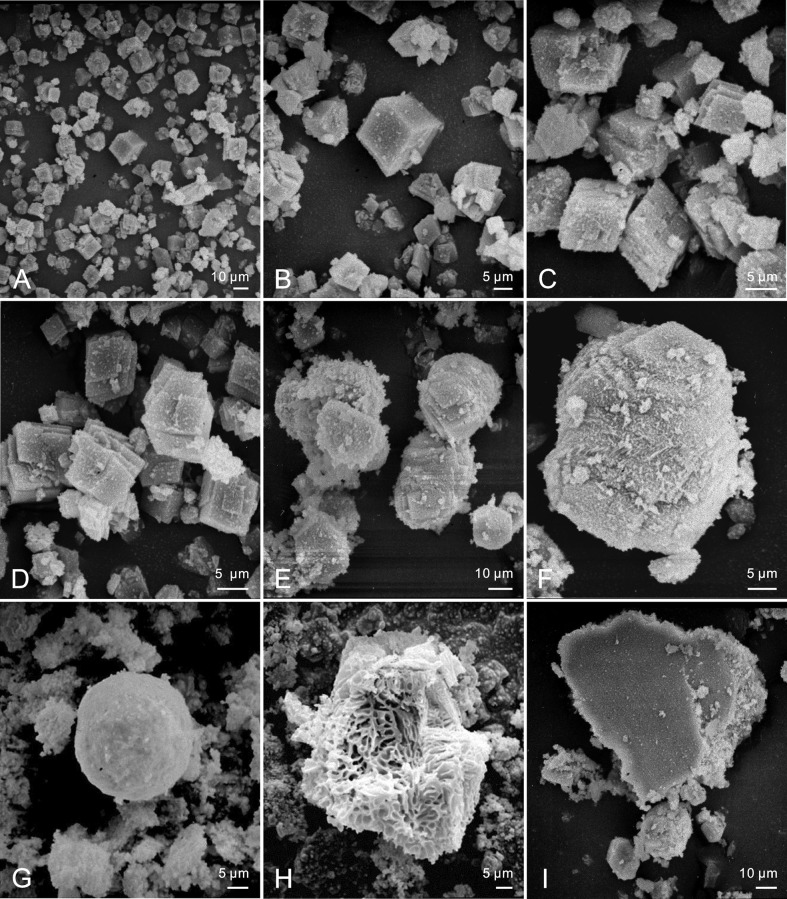
SEM micrographs revealing the different morphologies of calcite crystals induced by *B. lentus* CP28. A) morphologies of crystals; B) the cubic crystal; Cand D) the rhombic crystal; E and F) the polygonal plate-like caystal; G) the spherical crystal; H and I) the irregular crystal.

Morphologies of crystals induced by CP16, CP23 and CP28 were observed under SEM microscopy, and the results showed the morphologies of crystals induced by CP16, CP23 and CP28 were similar. Basically, five different morphologies of crystals, the cubic crystal, the rhombic crystal, the polygonal plate-like crystal, the spherical crystal, and the irregular shaped crystal, were observed (Figure 4). The cubic, the rhombic and the polygonal plate-like crystal were the three main shapes of crystals induced by the isolates, while the spherical and the irregular shaped crystals were less common (Figure 4A). Different morphologies of crystal showed the different properties of aggregated minerals described as follows. The surfaces of cubic shaped crystals were smooth (Figure 4B). The rhombic-shaped (Figure 4C and 4D) and the polygonal plate-like (Figure 4E and 4F) crystals generally presented well-defined faces and edges with accumulation of plate-like structures. The spherical crystals were formed by accumulation of granular composition with a rough surface (Figure 4G). The irregular porous shaped crystals appeared to be amorphous with many tiny holes inside (Figure 4H). Another kind of irregular crystals with smoothed surfaces were also occasionally observed (Figure 4I).

### Chemical process of calcium carbonate precipitation induced by *B. lentus* CP28

**Figure 5:**
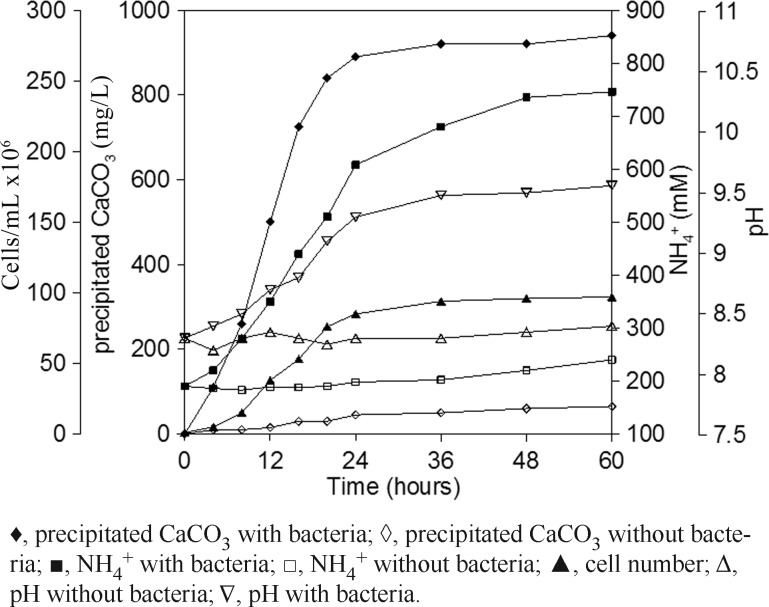
Dynamic analysis of calcium carbonate precipitation induced by *B. lentus* CP28 associated with changes of pH, ammonium ion, cell growth. Data reflect average of three experiments performed in triplicate.

Based on our observation and analyses, all the strains induced calcite precipitation in the liquid media. To determine the correlation of calcium carbonate formation with the metabolic parameter changes on the growth phase of *B. lentus* CP28, several parameters, including pH, cell number, ammonium ion concentration and mass of calcium carbonate, were monitored. The amount of calcium carbonate precipitation appeared to maintain a positive correlation with the growth of *B. lentus* CP28 (Figure 5). The pH quickly increased from the initial pH of 8.3 to 9.4 in the first 12 h of inoculation. While in the stage of log phase growth, *B. lentus* CP28 maintained robust growth, and the concentration of ammonium ions, which is believed to have contributed to the rise of pH, increased to 608 mM. Relatively more calcium carbonate precipitation was precipitated during this period. When the growth of *B. lentus* CP28 was in the stationary phase after 12 h of incubation, the pH gradually increased to 9.6 from 9.4 and the ammonium ion concentration slightly increased to 746 mM. During this period, the rate of calcium carbonate precipitation was lower than that of the first phase. The calcium carbonate precipitation tended to reach plateaus, with a production of 96 mg/L in this phase. However, in the control experiment without bacteria, the pH of the media increased only slightly from 8.3 to 8.4, and the concentration of ammonium ions remained relatively stable. With such slight alkalinity of the media, only trace calcium carbonate precipitation was collected in the control experiments (Figure 5).

## Discussion

Previous studies demonstrated that a great diversity of microbial genera took part in calcium carbonate precipitation in various natural environments (Wright and Oren, 2005), such as soils, freshwater, oceans and saline lakes (Douglas *et al.*, 1998; Rivadeneyra *et al.*, 1998; Peckman *et a*l., 1999; Zamarreño *et al.*, 2009). Our research showed that a variety of bacteria inhabiting the marine sediments could also induce calcium carbonate precipitation. Three genera of bacteria, *Sporosarcina*, *Bacillus* and *Brevundimonas*, were identified as precipitating calcium carbonate. *S. soli*, rather than *B. lentus* and *B. diminuta*, was found to be the dominant cultured species. In previous studies, *Sporosarcina pasteurii* (formerly known as *Bacillus pasteurii* from older taxonomies) and *Bacillus subtilis* were frequently reported to be isolated from various environments and studied for calcium carbonate biomineralization or for being a limestone consolidant (Stocks-Fischer *et al.*, 1999; Fujita *et al.*, 2000; Bachmeier *et al.*, 2002; Achal *et al.*, 2009; Zamarreño *et al.*, 2009; Okwadha and Li, 2010). *B. diminuta* was found to be the most effective carbonatogenic bacterium isolated from decayed building stones (Jroundi *et al.*, 2010; Rodriguez-Navarro *et al.*, 2012), while *B. lentus* from soil, marine waters and sediments was often used as a producer of alkaline protease in industry (Goddette *et al.*, 1992; Betzel *et al.*, 1988; Jørgensen *et al.*, 2000). Biomineralization of calcium carbonate facilited by *B. lentus* was also reported. Our data showed that the bacterium could strongly induce calcium carbonate precipitation in comparison to the other strains in our experiments. With considerable researches, broad ranges of bacteria were found to be involved in the process of calcium carbonate biomineralization. It is thought that calcium carbonate biomineralization is not necessarily linked to any particular group of organisms but rather a general phenomenon in the bacterial world (Boquet *et al.*, 1973; Ehrlich, 1998).

Considerable research on carbonate precipitation by bacteria has been performed using ureolytic bacteria (Hammes *et al.*, 2003; Ai-Thawadi, 2011), which by means of urea hydrolysis produce ammonia and carbonate ion, leading to an increase in pH, and thus favoring calcium carbonate precipitation. Our data showed that urease activity was present in all the isolates when tested on the urease activity assay media. The strain of *B. lentus* CP28 exhibited higher urease activity and more rapid growth and crystallization of calcium carbonate aggregation than strains *B. diminuta* CP16 and *S. soli* CP23. This observation coincides with the observations by Hammes *et al.* (2003), who reported a diversity of urease genes in the genomes of ureolytic bacteria and proposed that their high affinities and specific rates were the basase of rapid crystal formation. Urea is an organic nitrogenous compound present in coastal environments and introduced by the excretion of certain terrestrial and aquatic animals. Biotic urease activity is widespread in the environment and includes the actions of bacteria, yeasts and filamentous fungi (Mobley and Hausinger, 1989). Urease hydrolyses the substrate urea, which creates an alkaline environment to facilitate calcium carbonate precipitation in the natural settings, and thus partially contributes to the marine lithifications. With removal of urea from the experimental media, strains *B. diminuta* CP16 and *S. soli* CP23 were switched to metabolizing glucose and probably produced organic acids to dissolve calcium carbonate (Table 1). This phenomenon was observed in cave isolates, demonstrating their abilities to precipitate and dissolve calcium carbonate (Banks *et al.*, 2010). Therefore, we inferred that the processes of both precipitation and dissolution of calcium carbonate are dynamic processes in the natural marine sediment system, with both processes depending on the availability of urea and other organic substances.

Calcite, aragonite and vaterite are three crystalline polymorphs of calcium carbonate existing in natural environments. Calcite and vaterite are the most common crystalline polymorphs induced by ureolytic bacteria (Boquet *et al.*, 1973; Rivadeneyra *et al.*, 1996; Stocks-Fisher *et al.*, 1999; Bang *et al.*, 2001; Giralt *et al.*, 2001; Bachemeier *et al.*, 2002; Kawaguchi and Decho, 2002;). It has been reported that aragonite could also be precipitated by bacteria (Rivadeneyra *et al.*, 1996). Zamarreño *et al.* (2009) demonstrated that *Pseudomonas* D2 and F2 have a remarkable ability to induce the precipitation of primarily calcite and vaterite, similar to the results obtained by Rodriguez-Navarro *et al.* (2003) and Muynck *et al.* (2008), who used *Myxococcus xanthus* and *Bacillus sphaericus* in their work*,* respectively. *Acinetobacter* B14 induced more precipitation of vaterite than calcite (Zamarreño *et al.*, 2009), *Deleya halophlia* induced precipitation of aragonite (Rivadeneyra *et al.*, 1996), whereas *Lysinibacillus sphaericus* INQCS 414 precipitated only vaterite (Shirakawa *et al.*, 2011). In contrast, our research isolates of *B. diminuta* CP16, *S. soli* CP23 and *B. lentus* CP28 predominantly induced calcite precipitation. Nevertheless, this finding is consistent with the results from Li *et al.* (2011) and Achal *et al.* (2009), who reported using *Bacillus sp.* and *S. pasteurii* MTCC 1761, respectively, to induce calcium carbonate precipitation. Despite extensive studies on bacterial carbonatogenesis, little is known about what causes bacteria to precipitate different carbonate polymorphs. Besides the observation that the particular bacterial species used has an important influence on the type of carbonate precipitation, the composition of the culture medium is also believed to be one of the determinants (Rivadeneyra *et al.*, 1996, 1998; Gonzalez-Munoz *et al.*, 2010;). It has also been reported that the specific amino acid sequence in the urease enzyme of bacteria may be responsible for the carbonate polymorph selection. Higher concentration of Asp and Glu in the urease of *B. pasteurii* favored the formation of vaterite, while calcite was the predominant precipitate when using urease of *Canavalia ensiformis* with a lower concentration of Asp and Glu (Sondi and Salopek-Sondi, 2005). Kawaguchi and Decho (2002) reported that specific proteins in extracellular polymeric substances (EPS) of *Schizothrix sp*. influenced aragonite and calcite polymorph selection. These previous studies suggest that the polymorph selection is a complex process involving a variety of abiotic and biotic factors.

Our results showed that *B. diminuta* CP16, *S. soli* CP23 and *B. lentus* CP28 induced similar morphologies of crystals. The cubic, rhombic and polygonal plate-like crystals were the dominant crystals compared with the less common spherical and irregularly shaped crystals. This result is consistent with that Tourney and Ngwenya (2009) observed from a strain of *Bacillus licheniformis* S-86. According to Li *et al.* (2010), the crystal morphologies of precipitates produced by *Bacillus sp*. mainly showed cubic and polyhedral shapes. Zamarreño *et al.* (2009) reported that microbial calcite crystals presented a variety of morphologies depending on the type of isolate. *Pseudomonas putida* F2 induced nailhead and spheroidal crystals, *Pseudomonas aeruginosa* D2 induced pseudo-ellipsoidal and pseudo-cubic crystals, whereas *Acinetobacter junii* B14 induced semi-spheroidal, pseudo-hexagonal prism and nailhead spar crystals in the same growth medium.

The process of calcium carbonate precipitation is usually governed by four key factors: (1) calcium concentration, (2) concentration of dissolved inorganic carbon, (3) pH, (4) the availability of nucleation sites (Muynck *et al.*, 2010). The first three factors can be influenced by bacteria, most notably, the creation of an alkaline environment. Moreover, the bacteria can also provide the crystal nucleation sites for calcium carbonate precipitation (Hammes and Verstraete, 2002). In the process of calcium carbonate precipitation, bacterial precipitation caused faster precipitation rates than chemical precipitation (Stocks-Fischer *et al.*, 1999). As shown in Figure 5, calcium carbonate precipitation was clearly correlated with the growth of *B. lentus* CP28, which utilized ureases to hydrolyze urea and generate carbonate and ammonia, and result in an increase in pH. With the rise of pH, more ammonium ion was released and a considerable quantity of calcium carbonate was precipitated. On the other hand, it is also possible that with the bacterial cell number increase more crystal nucleation sites were available, favoring the calcium carbonate precipitation (Stocks-Fisher *et al.*, 1999). In the bacterium-free control, both pH and ammonium ion concentration kept increasing slightly while little calcium carbonate precipitation was collected. The kinetics of microbial calcium carbonate precipitation are similar to those of reported by Stocks-Fisher *et al.* (1999). The data obtained from the bacterial process of calcium carbonate precipitation induced by *B. lentus* CP28 provides straightforward evidence to understand microbial calcium carbonate precipitation.

In summary, in this paper we have clearly shown that three species of bacteria isolated from marine sediment participate in microbial calcium carbonate precipitation through hydrolysis of urea. Mineralogical analysis of the induced calcium carbonate precipitation shows that calcite is the dominant carbonate polymorph, and morphologies of crystals are mainly cubic and rhombic. These results suggest that production of carbonate polymorph is not specifically related to any bacterial species, but is rather influenced by complicated environmental factors such as the pH, the composition of the media, etc.
